# Understanding the margin squeeze: Differentiation in fitness‐related traits between central and trailing edge populations of *Corallina officinalis*

**DOI:** 10.1002/ece3.5162

**Published:** 2019-05-07

**Authors:** Regina Kolzenburg, Katy R. Nicastro, Sophie J. McCoy, Alex T. Ford, Gerardo I. Zardi, Federica Ragazzola

**Affiliations:** ^1^ Institute of Marine Sciences University of Portsmouth Portsmouth UK; ^2^ Centre of Marine Sciences (CCMAR) University of Algarve Faro Portugal; ^3^ Department of Biological Sciences Florida State University Tallahassee Florida; ^4^ Department of Zoology and Entomology Rhodes University Grahamstown South Africa

**Keywords:** calcification, climate change, common garden experiment, coralline algae, intertidal, photosynthesis, P‐I curve, uncoupling

## Abstract

**Abstract:**

Assessing population responses to climate‐related environmental change is key to understanding the adaptive potential of the species as a whole. Coralline algae are critical components of marine shallow water ecosystems where they function as important ecosystem engineers. Populations of the calcifying algae C*orallina officinalis* from the center (southern UK) and periphery (northern Spain) of the North Atlantic species natural distribution were selected to test for functional differentiation in thermal stress response. Physiological measurements of calcification, photosynthesis, respiration, growth rates, oxygen, and calcification evolution curves were performed using closed cell respirometry methods. Species identity was genetically confirmed via DNA barcoding. Through a common garden approach, we identified distinct vulnerability to thermal stress of central and peripheral populations. Southern populations showed a decrease in photosynthetic rate under environmental conditions of central locations, and central populations showed a decline in calcification rates under southern conditions. This shows that the two processes of calcification and photosynthesis are not as tightly coupled as previously assumed. How the species as whole will react to future climatic changes will be determined by the interplay of local environmental conditions and these distinct population adaptive traits.

**OPEN RESEARCH BADGES:**

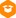

This article has earned an Open Materials Badge for making publicly available the components of the research methodology needed to reproduce the reported procedure and analysis. All materials are available at https://doi.pangaea.de/10.1594/PANGAEA.899568.

## 1 INTRODUCTION

Anthropogenic input of carbon dioxide in the atmosphere resulted in a changing climate over the past decades and might further fuel global climate change in future centuries (IPCC, 2013). Key components of climate change in the ocean are ocean acidification (OA; decreasing pH and Ω${\text{CO}}_{3}^{2-}$) and rising sea surface temperatures. Living organisms already experience effects of climatic changes and will continue to be critically affected by these changes (Bentz et al., 2010; Lesica & McCune, 2004; Sanford, Holzman, Haney, Rand, & Bertness, 2006). Investigating and monitoring organism physiology provides important information to predict their acclimatization and resilience to future environmental conditions and potential changes in their distribution (Kelly & Hofmann, 2013).

It is suggested that the geographic center of a species distribution holds the most favorable conditions and therefore holds the highest population density (Brussard, 1984; Whittaker, 1956). When moving away from the center toward the margins of the distribution, environmental variables are thought to become less favorable due to greater abiotic stress and increased interspecific competition (Aitken, Yeaman, Holliday, Wang, & Curtis‐McLane, 2008), initiating a decrease in population densities and lower relative fertility (Case & Taper, 2000). Further, Watkinson and Sutherland (1995) described a higher mortality than recruitment rate for local marginal populations, also called sink populations, whose survival depends on influx of zygotes or spores from source populations. Species react differently to environmental changes. In terrestrial studies, Franco et al. (2006) showed that three out of four butterfly species are reported extinct or showed drastic distributional changes due to climate change along with habitat degradation and loss. Another study by Lesica and McCune (2004) showed that the southern margin of arctic–alpine indicator plants species declined in their abundance within 13 years which is caused by increasing average summer temperatures. Previous research has also found that different populations of the same species respond differently to altered environmental conditions (Bozinovic, Calosi, & Spicer, 2011; Calosi et al., 2017; Gaston, 2009). In the marine environment, a number of studies across several taxonomic groups, for example, fish, molluscs, zooplankton, or seaweed, have focused on the diversification of populations along a thermal‐latitudinal gradient (Bennett, Wernberg, Arackal Joy, Bettignies, & Campbell, 2015; Dam, 2013; Lucassen, 2013; Morley, Hirse, Pörtner, & Peck, 2009).

Calcifying organisms are at the forefront of those affected by climatic changes and can therefore act as indicator species for induced impacts on marine organisms. One of the major groups effected by climate change is calcifying benthic macroalgae (Kroeker et al., 2013). Amongst those are coralline red algae (Corallinales, Rhodophyta) which are critical components of marine shallow water ecosystems from polar regions to the tropics (Adey & MacIntyre, 1973; Steneck, 1986). They function as important ecosystem engineers and play a crucial role as an essential structural element in the majority of rocky coastal zones (Benedetti‐Cecchi, 2006; Dayton, 1972; van der Heijden & Kamenos, 2015; Johansen, 1981; Jones, Lawton, & Shachak, 1994; Kelaher, Chapman, & Underwood, 2001; Nelson, 2009; Noël, Hawkins, Jenkins, & Thompson, 2009). They often form complex, extremely dense, and highly branched turfs which are considered the extreme end of algal structural complexity (Coull & Wells, 1983; Davenport, Butler, & Cheshire, 1999). Branches consist of calcified segments (intergenicula), which are produced through high‐magnesium (Mg) calcite precipitation in the cell walls, and noncalcified segments (genicula); this structure provides flexibility and elasticity for every individual branch (Martone & Denny, 2008). Coralline turf is highly variable, with frond length and density differing at small spatial scales; they can host abundant and diverse macrofaunal assemblages with up to 250,000 individuals per m^2^ (Kelaher et al., 2001). In addition, macroalgal physiological processes such as photosynthesis and respiration alter CO_2_ and ${{\mathrm{HCO}}_{3}}^{-}$ in the water in the intertidal environment. This causes changes in pH across diurnal as well as spatial scales related to species distributions (Morris & Taylor, 1983; Williamson et al., 2014; Williamson, Perkins, Voller, Yallop, & Brodie, 2017). Depending on the extent of these changes in the carbonate chemistry and especially in combination with the climatic changes named above, calcification of coralline algae can be severely affected.

Geniculated coralline algae (also known as articulated coralline algae), like *Corallina officinalis*, form turfs across large areas on hard substratum in the intertidal ecosystems of the Northeast Atlantic. *C. officinalis* is commonly found in sheltered, low intertidal zones, where it primarily inhabits the lower part of rock pools and channels that remain damp or filled during extreme tides or conditions, and at the edge of the intertidal to subtidal zones (Digby, 1977; Egilsdottir, Noisette, Noel, Olafsson, & Martin, 2013; Williamson et al., 2014). In order to maintain their abundance in temperate intertidal ecosystems, coralline algae are suggested to have a good ability to adapt to great and fast changes in environmental conditions, such as solar irradiance, physical stress, water temperature, or carbonate chemistry (e.g., large pH variations) which fluctuate tidally, diurnal, monthly, and seasonally (Egilsdottir et al., 2013; Hofmann et al., 2014; Martone, Alyono, & Stites, 2010; Williamson et al., 2014).

In particular, temperature is one of the main factors governing the small‐scale vertical distribution of macroalgae on a shore (Lüning, 1990) and the large‐scale geographical distribution of macroalgal species (Ganning 1971). At the organism level, temperature regulates major chemical reactions, which in turn affect metabolic pathways (Lobban & Harrison, 1994). For example, carbonic anhydrase (CA) is affected by temperature altering the carbon fixation pathways in photosynthesis (Lobban & Harrison, 1994). Water temperature affects recruitment, survival, growth as well as reproduction of macroalgae (Breeman, 1988) and thus drives the species’ distribution (Breeman, 1988; Lüning, 1990). Most importantly, current increase in global temperature, and therefore a possible exceeding of the species’ temperature threshold, is causing species‐level responses in macroalgae, such as species range shifts (reviewed by Helmuth, Mieszkowska, Moore, & Hawkins, 2006; Nicastro et al., 2013; Smale & Wernberg, 2013; Parmesan, 2006; Wernberg et al., 2011). This ongoing temperature increase, and what is predicted for future scenarios, is causing a chronic, due to gradual warming, or an acute stress, due to extreme temperature events (Brodie et al., 2014). Adaptations will need to include facilitation of all metabolic processes at elevated temperatures, especially those for photosynthesis, respiration, calcification, and therefore growth. Hofmann Straub and Bischof (2012), Hofmann, Yildiz, Hanelt, and Bischof (2012), Egilsdottir et al. (2013), and Noisette et al. (2013) found that with ongoing climate change, and therefore worsening OA and rising water temperatures, interactions between coralline algal physiology and variable environmental parameters are likely to be significantly negatively affected.

It remains unclear how and whether the wider distributed, turf‐forming algae *C. officinalis* will be able to withstand such changes. It is likely that the species as a whole will show some resilience when exposed to predicted climate change conditions. However, it is uncertain which areas or portions of the species distribution will be affected the most by the changes. At present, there are very few studies regarding the physiological responses of intertidal benthic organisms to current climate conditions across its distribution (reviewed by Helmuth et al., 2006; Díez, Muguerza, Santolaria, Ganzedo, & Gorostiaga, 2012). This gap in knowledge complicates the establishment of detailed predictions for this species future (Brodie et al., 2014; Nelson, 2009; Williamson et al., 2017). In this study, physiological responses (photosynthesis, respiration, calcification, photosynthesis–irradiance curves, and calcification–irradiance curves) of *C. officinalis* across its natural distribution in the eastern North Atlantic were investigated using the approach of common garden experiments. England was chosen as central population for this study, referring to confirmed genetic data by Brodie, Walker, Williamson, and Irvine (2013). Spain was studied representing the southern margin of the species distribution (Williamson et al., 2015).

We hypothesized that central populations are more robust to elevated temperatures and therefore are able to adapt to the temperature conditions already experienced by southern populations. In contrast, we predicted that southern populations will be able to adapt to the most favorable central conditions.

## 2 MATERIAL AND METHODS

### 2.1 Species collection

Specimens (*n* = 72) of *Corallina officinalis* Linneaus populations (*n* = 2) were collected intertidally, during low tide in an average depth of 0.31m from the water surface at sites in the South East Coast of the UK (central population 1: St Margarets Bay: N 51.148056, E 1.385056; population 2: Westbrook Bay: N 51.388840, E 1.367170; beeline distance between populations: 26.85 km) and the North West Coast of Spain (southern margin population 1: Illa del Arousa: N 42.56870, W 8.89171; population 2: Tragove: N 42.52444, W 8.82772; beeline distance between populations: 7.19 km) in February 2017. Specimens were transported into the laboratory at the Institute of Marine Sciences of the University of Portsmouth, UK, using temperature‐insulating containers. Only healthy specimens without epiphytes and indication of bleaching or damage were selected for this study.

### 2.2 Species identification

To verify species identification, genomic DNA was extracted from three replicates of each population using PowerSoil DNA Isolation Kit (MoBio Laboratories, Carlsbad, CA) in accordance with the manufacturer's instructions. Partial amplification of around 664‐bp fragment of standard DNA barcode region (COI‐5P) was performed using primers GazF1 and GazR with polymerase chain reaction conditions described in Pardo, Peña, Berreiro, and Bárbara (2015). Amplified fragments were run in an ABI PRISM 3130xl automated capillary sequencer (Applied Biosystems, CCMAR Portugal). MtDNA sequences were aligned, proofread, and edited in GENEIOUS 3.8 (Drummond et al., 2011). Determination of taxonomic status was made by comparing the sequences obtained in our study to published sequences retrieved from GenBank. For *Corallina officinalis*: BM000806013, BM000804477, BM000804459, BM000804370, BM000804472 (Williamson et al., 2015). For *Ellisolandia elongata*: JQ615843, BM000806006 (Williamson et al., 2015), KF461004, KF461018, KF461017 (Pardo et al., 2015). For *Corallina caespitosa*: BM000806012, BM000804521, BM000804492, BM000804378, BM000806003 (Williamson et al., 2015). Following an alignment and trimming of GenBank sequences and the ones from this study, pairwise sequence similarity scores were calculated using CLUSTALW multiple sequence alignment program (Thompson, Higgins, & Gibson, 1994; Table 1).

**Table 1:** Pairwise sequence similarity scores (%)

	*Corallina officinalis*—this study
*Corallina officinalis*—Williamson et al. (2015)	99.59–100
*Corallina caespitosa*—Williamson et al. (2015)	89.79–92.24
*Ellisolandia elongata*—Williamson et al. (2015) & Pardo et al. (2015)	83.67–84.89

### 2.3 Experimental setup

**Figure 1:**
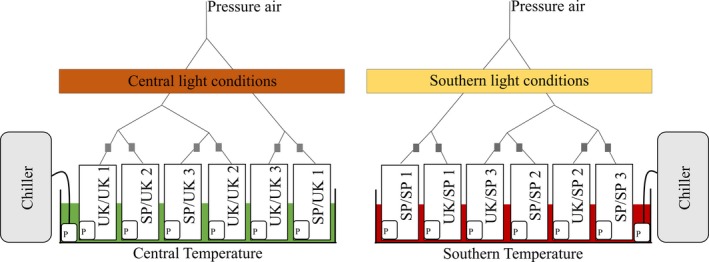
Detailed schematic of the common garden experiment setup. Representing central (UK) conditions on the left in green and dark orange and southern (Spanish, SP) conditions on the right in red and light orange. Squares with “P” represent installed pumps for water movement. Gray squares represent pressure air adjustment valves

In the laboratory, specimens were carefully placed upright onto a rock in the water, held in place with a net (2 mm mesh size) in order to simulate natural conditions and each population was color coded for future identification. Aquaria of 11.1 L (*n* = 3) for each of the two countries and temperature conditions (total *n* = 12) were set up in a water bath (for detailed setup see Figure 1). 75% of the water in the replicate aquaria was changed every second day and treated with UV light (P2‐110W Commercial UV Steriliser, max. flow rate: 65 L/min. Tropic Marine Centre (TMC), London, UK) prior to the disposal. Temperature conditions were kept constant using TK‐2000 chillers (cooling capacity of 870 W, 800 L/hr. TECO, Ravenna, Italy). Each aquarium was equipped with a water pump (V^2^PowerPump 800, Flow rate: 700 L/hr. TMC, London, UK) and an air stone to simulate tidal water movements and ensure oxygen supply with ambient air. Specimens were kept at their respective local in situ temperatures measured during sampling (central conditions: temperature: 5.7°C, light: 57 μmol m^−2^ s^−1^; southern conditions: temperature: 11.3°C, light: 184 μmol m^−2^ s^−1^). Both treatments were subject to a light:dark cycle of 10:14 hr (taken from the average of natural conditions of center and southern locations) produced and controlled by AQUABEAM 600 Ultima Reef White lights with cloud function (TMC, London, UK). Additionally, sunset and sunrise were mimicked through a slow increase and decrease of light intensities over a period of 1 hr. All specimens were kept in their corresponding conditions for a minimum of 1 week to acclimate to aquaria conditions. Following the initial acclimatization, half of the central and southern populations were gradually acclimatized (increase/decrease of 1.5°C/week and 40 μmol m^−2^ s^−1^/week, respectively) to the opposite temperature and light conditions over a period of 3.5 weeks. After acclimatization, specimens were randomly and equally distributed within the corresponding replicate aquaria and kept for three months (Figure 1).

### 2.4 Monitoring of water parameters

Temperature, salinity, dissolved oxygen, pH, and total alkalinity (A_T_) were monitored daily. Irradiance was measured once a month to monitor the decrease in light intensity using a HOBO UA‐002‐64 Pendant Temp/Light data logger (accuracy: 0.47°C, resolution: 0.1°C. Tempcon, Arundel, UK). Temperature and salinity were measured with a CO310‐1 portable salinity and temperature probe (accuracy: 0.2% for salinity, ±0.2°C for temperature; resolution: 0.1 for salinity, 0.1°C for temperature. VWR, Leicestershire, UK). Dissolved oxygen and pH were measured with an HQ30d portable multi‐parameter meter and a luminescent dissolved oxygen (LDO101, accuracy: ±0.1 mg/L. HACH, Manchester, UK) and pH probe (PHC301, accuracy: ±0.02 pH, HACH, Manchester, UK. Calibrated on the National Bureau of Standards (NBS) scale and converted into total scale values using Tris/HCl and 2‐aminopyridine/HCl buffer solutions after Dickson, Sabine, and Christian (2007)). A_T_ was monitored daily in one randomly chosen replicate for each treatment, using the alkalinity anomaly technique after Smith and Key (1975), Chisholm and Gattuso (1991), and Dickson et al. (2007), (SOP 3b). A_T_ titrations were carried out with an automatic titrator TitroLine 7000 (measurement accuracy: 0.002 ± 1 digit, dosing accuracy: 0.15%, dosing precision: 0.05%–0.07%. Schott SI Analytics, Mainz, Germany) to ensure minimal variation. As titrant, 0.1N hydrochloric acid (HCl) was used, which was validated against Certified Reference Material provided by Andrew G. Dickson (Batch 154, Scripps Institution of Oceanography).

### 2.5 Physiological incubation procedures and measurements

To determine saturating light levels of *C. officinalis* populations before and after the experiment, the oxygen production (P‐I curve) and calcification (C‐I curve) evolution under increasing light conditions were measured. For this, 0.97 ± 0.07 g fresh weight of algal fronds was transferred into a clear closed cell 58 ml incubation chamber and incubated successively for 1 hr at each of the seven different light intensities (0, 20, 80, 160, 320, 500, and 700 μmol m^−2^ s^−1^; AQUARAY Nature Perfect, TMC, London, UK), starting from the lowest to the highest to minimize stress. Three replicates per light intensity were incubated simultaneously and bubble‐free. To account and correct for metabolism effects by other organisms, reference incubations without algae were performed alongside the above‐described incubations for all light intensities. Aluminum foil was used to coat the chambers to determine calcification and respiration rates in the dark. Irradiance was measured with a Quantitherm PAR/Temperature Sensor with a QTP1 probe (resolution: 1 μmol m^−2^ s^−1^, 0.02°C, respectively. Hansatech, Norfolk, UK).

Differences in oxygen concentration, pH, and A_T_ were determined in order to calculate photosynthesis, respiration, and calcification rates by measuring oxygen, pH, and A_T_, respectively, before and after the incubations. Prior to the A_T_ measurements, seawater samples were filtered through a syringe filter (hydrophilic 25 mm, 0.2 μm PTFE. Fisherbrand, Loughborough, UK) into sterile 50 ml tubes (Plastic Centrifuge Tubes. Fisherbrand, Loughborough, UK). Immediately after, potentiometric titrations were performed using an automatic titrator (details: see above) calibrated on the NBS scale. Results were calculated based on a Gran function after Dickson et al. (2007). The carbonate system of the seawater was calculated from pH, A_T_, salinity, and temperature using the Excel Macro CO2Sys (Version 2.1, Lewis & Wallace, 1998) with K1 and K2 according to Mehrbach, Culberson, Hawley, and Pytkowicx (1973) and refit by Dickson and Millero (1987) (Table 2). Measurement ranges of pH values represent in situ values and are highly dependent on temperature.

**Table 2:** Carbonate system parameters of the whole setup during the 3‐month culturing of Corallina officinalis. All numbers are mean values (*n* = 81 ±*SD*). pH, salinity, temperature, and total alkalinity (A_T_) were measured while the other parameters were calculated

Treatment	pH (total scale)	Sal	T [°C]	A_T_ [μmol/kg]	DIC [μmol/kg]	HCO_3_^‐^ [μmol/kg]	Ω_Ca_	Ω_Ar_	pCO_2_ [μatm]
Center	8.19 ± 0.07	32.9 ± 0.6	5.70 ± 0.73	2,344 ± 43	2,185.9 ± 46.6	2049.1 ± 50.6	2.32 ± 0.27	1.48 ± 0.17	377 ± 61
South	8.22 ± 0.08	33.2 ± 0.5	11.33 ± 0.39	2,326 ± 37	2,100.0 ± 46.4	1925.6 ± 54.3	3.23 ± 0.30	2.08 ± 0.19	319 ± 45

To determine changes in photosynthesis, respiration, and calcification rates most likely to be found in the field, additional incubations (*n* = 3) of all treatments at ambient light conditions (57 μmol m^−2^ s^−1^ for center and 184 μmol m^−2^ s^−1^ for southern populations) were performed before and after the study following the protocol above. Calculations were performed following the methodology described above.

The weight of algae fronds used in the incubations was transformed from FW into dry weight (DW) by multiplying FW measurements with the factor 0.62286. This factor was determined from FW and DW weight measurements of thirty 1 g frond bundles from all populations before and after algae were left to dry for 48 hr in an oven at 60ºC.

### 2.6 Data and statistical analysis

The maximum potential photosynthetic rate per individual (*P*_max_ [mg O_2_ g^−1^ FW hr^−1^]) was directly determined from the oxygen evolution curves, whereas the slope of the light‐limited region of the P‐I curve stating the efficiency of the algae to harvests light (α) was calculated using Excel. The irradiance at which photosynthesis is saturated (*I*_K_ [μmol m^−2^ s^−1^]) was calculated using the following relationship:

(1) $$I_{k}=\frac{P_{\max}}{\alpha }$$

Primary production was measured using oxygen fluxes such as photosynthesis (*P*_N_ [mg O_2_ g^−1^ FW hr^−1^]) and respiration (R [mg O_2_ g^−1^ FW hr^−1^]) and was calculated as follows:

(2) $$P_{N}\;\text{or}\;R\left(O_{2}\right)=\left(\frac{\Delta O_{2}\times V}{\Delta t\times \text{FW}}\right)$$

with ∆O_2_ as the change in dissolved oxygen concentration [mg/L], V as the volume of the incubation chamber [L], ∆*t* as the incubation time [hr], and FW as the fresh weight of the algae.

Calcification (*G* [meq CaCO_3_ g^−1^ FW hr^−1^]) rates were estimated using the following equation:

(3) $$G\left({\text{CaCO}}_{3}\right)=-\left(\frac{\Delta T_{A}\times V}{2\times \Delta t\times \text{FW}}\right)$$

with ∆*A*_T_ as the change in total alkalinity [meq/L].

All analyses were run in SPSS Statistics 24 (IBM Corp, 2016), and data were tested for normality and homogeneity prior analyses (Shapiro–Wilk and Levene's test, for *p* values, Table S1, see Data Repository). Data for net production (*n* = 3) were not normally distributed, resulting in statistical analysis using the nonparametric Kruskal–Wallis *H* test. Data for respiration (*n* = 3) as well as dark calcification (*n* = 3) measurements showed a significant Levene's test with raw as well as transformed data due to high variance. Therefore, data were analyzed using multiple single comparisons of one‐way analysis of variance (ANOVA). Normally distributed data of the dependant variable light calcification (*n* = 3) were analyzed using two‐way ANOVAs for individuals from each country of origin (UK (center) or SP (southern margin)) with treatment (three levels: pre‐experiment, central conditions, and southern conditions) as the fixed factor. When significant effects were found, data were further explored by running a post hoc Tukey's HSD test. Tests were performed for central and southern populations under each temperature condition comparing measurements before and after the experiment.

Additional two‐way ANOVAs and Tukey's HSD post hoc tests of central and southern populations were performed to identify significant differences in their maximum oxygen production and calcification level of all populations in all temperature treatments.

## 3 RESULTS

The experimental setup allowed for very stable parameters under all conditions and was not unexpectedly influential toward physiological behavior of specimens. All samples were identified as *C. officinalis*. Sequences have been submitted to Gene Bank (accession numbers central populations: BankIt2159662 COUK1 MK072600 – BankIt2159662 COUK6 MK072605 and southern populations: BankIt2159662 COSP1 MK072606 ‐ BankIt2159662 COSP6 MK072611).

### 3.1 P‐I and C‐I curves

**Figure A1:**
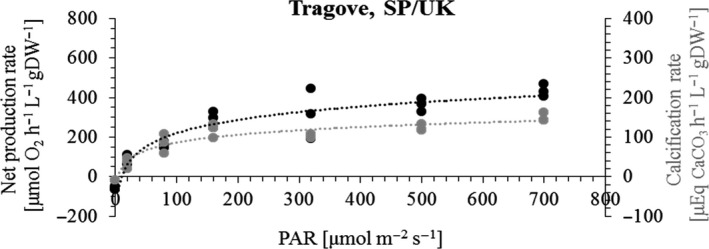
Oxygen and calcification evolution curve of the Tragove (southern) population under central conditions. Black plot refers to net production data. Gray plot refers to calcification rates

**Figure A2:**
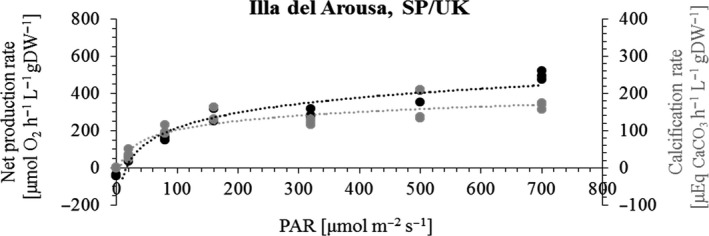
Oxygen and calcification evolution curve of the Illa del Arousa (southern) population under central conditions. Black plot refers to net production data. Gray plot refers to calcification rates

**Figure A3:**
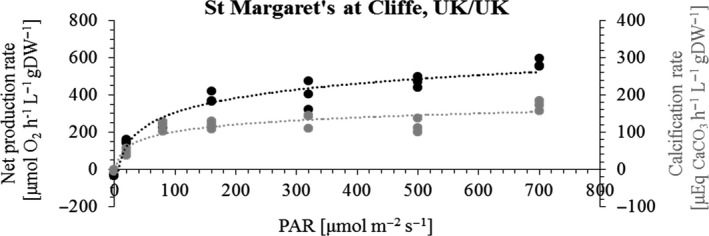
Oxygen and calcification evolution curve of the St Margaret's at Cliffe (center) population under central conditions. Black plot refers to net production data. Gray plot refers to calcification rates

**Figure A4:**
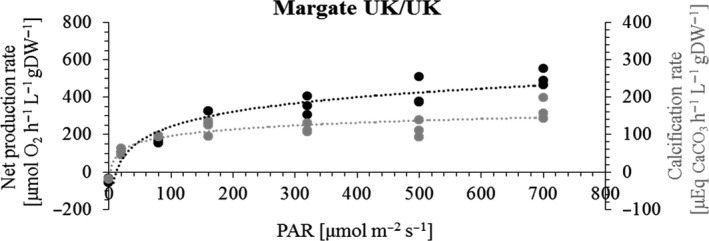
Oxygen and calcification evolution curve of the Margate (center) population under central conditions. Black plot refers to net production data. Gray plot refers to calcification rates

**Figure A5:**
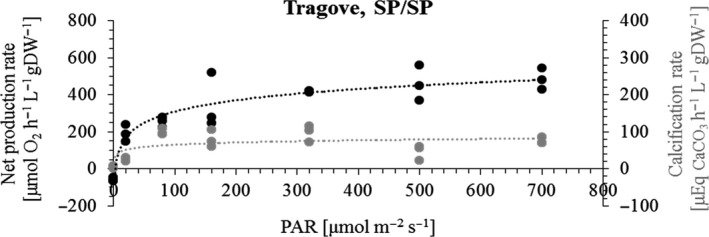
Oxygen and calcification evolution curve of the Tragove (southern) population under southern conditions. Black plot refers to net production data. Gray plot refers to calcification rates

**Figure A6:**
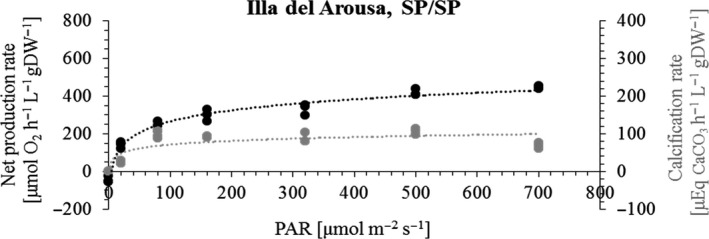
Oxygen and calcification evolution curve of the Illa del Arousa (southern) population under southern conditions. Black plot refers to net production data. Gray plot refers to calcification rates

**Figure A7:**
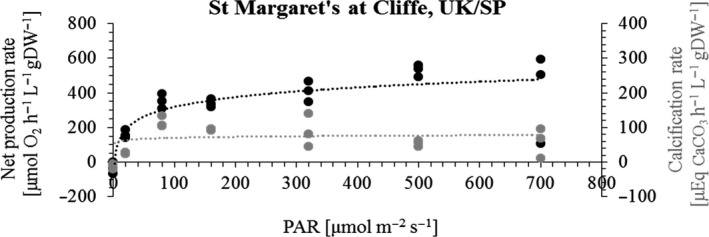
Oxygen and calcification evolution curve of the St Margaret's at Cliffe (center) population under southern conditions. Black plot refers to net production data. Gray plot refers to calcification rates

**Figure A8:**
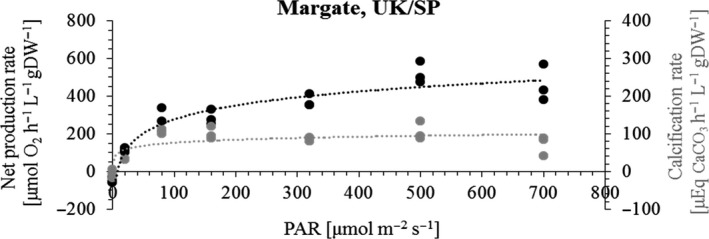
Oxygen and calcification evolution curve of the Margate (center) population under southern conditions. Black plot refers to net production data. Gray plot refers to calcification rates

The average saturating light levels (*P*_max_) for all populations of one country did not differ between central or southern temperature conditions after 3 months of culturing (Figure 1; Figures A1–A8 in Appendix A). *P*_max_ of central populations was ~1.8 and ~1.2 times higher than of southern populations under southern and central conditions, respectively. Central population 1 showed a significant difference versus southern populations under central conditions (*p* < 0.05, Tukey HSD *p* < 0.05, 2‐way ANOVA in both cases). Southern population 1 showed a significant difference versus central populations under southern conditions (*p* < 0.05, Tukey HSD *p* < 0.05, 2‐way ANOVA in both cases). The photosynthetic efficiency, α, of central populations did not differ in both treatments. However, all southern populations under central conditions showed up to 50% less light harvesting efficiency than under southern conditions. The saturating light intensity *I*_K_ of all populations was noticeably higher in central than in southern populations, under southern conditions, but very similar under central conditions. Southern populations developed a ~50% higher saturating light intensity in central (~85 μmol m^−2^ s^−1^) than under southern conditions (~44 μmol m^−2^ s^−1^).

**Figure 2:**
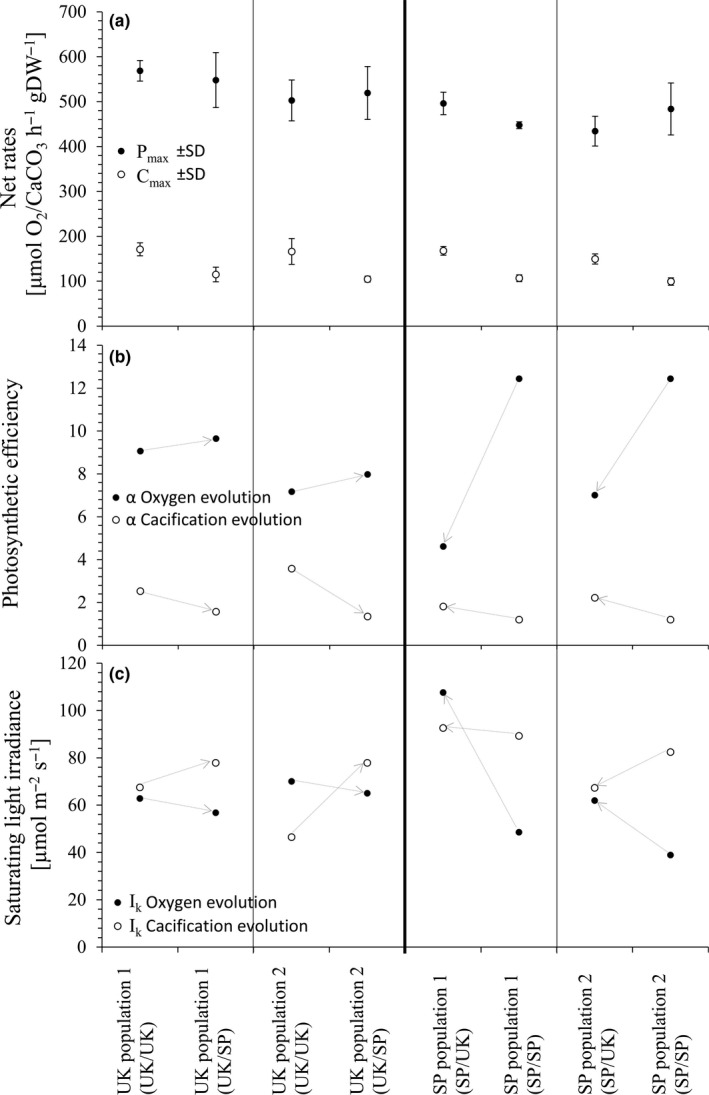
Evolution curve characteristics for net production and calcification (AV ± *SD*,* n* = 3) of the central (UK) and southern (SP) populations (*n* = 2) under both southern (SP) and central (UK) conditions. (a) *P*_max_/*C*_max_ = maximum production, (b) *α* = initial slope as indicator for photosynthetic efficiency, (c) *I*_K_ = saturating irradiance. Arrows indicate direction of change from original to altered conditions. For detailed graphs, see Figures A1–A8 in Appendix A. Statistical differences are summarized in Table S1 of the supplementary document

The average saturating calcification levels (*C*_max_) were ~1.54 times significantly higher under central compared to southern conditions in all four populations (*p* < 0.005, Tukey HSD *p* < 0.005, 2‐way ANOVA for all four cases) (Figure 2; Figures A1–A8 in Appendix A). The calcification efficiency *α* was higher under central than under southern conditions. Central populations showed a slightly higher efficiency than southern populations under both temperature conditions (Figure 2). Central populations showed a decrease in efficiency under southern conditions by a factor of 1.6 compared to central conditions. Southern populations showed an increasing trend by a factor of ~1.68 in light harvesting efficiency in central compared to southern populations. The saturating light intensity *I*_K_ did not change drastically in southern populations when acclimatized to southern or central conditions. In central populations, however, *I*_K_ increased by 132.5% (~19 μmol m^−2^ s^−1^) under southern conditions in comparison to central conditions.

Photoinhibition of photosynthesis rates was not observed in any of the populations; *P*_max_ was reached in all populations and treatments. Inhibition of calcification was observed in all populations under southern conditions.

### 3.2 Photosynthesis and respiration

**Figure 3:**
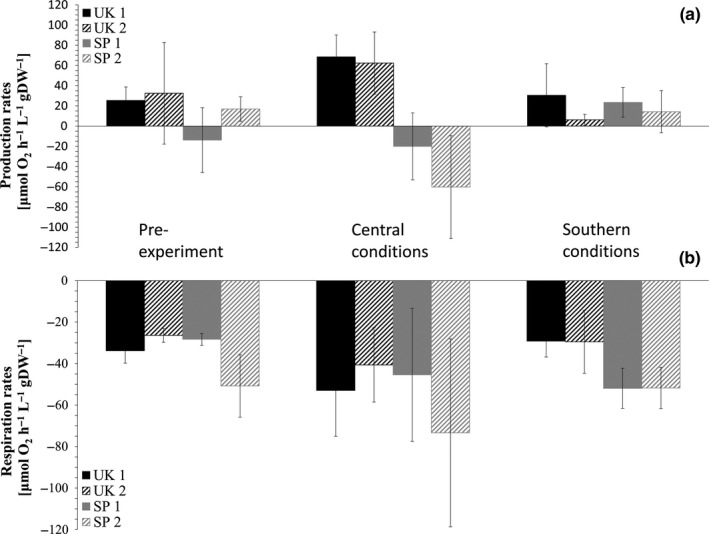
(a) Primary production and (b) respiration rates [μmol hr^−1^ L^−1^ gDW^−1^] ± *SD* of *Corallina officinalis* before and after the common garden experiment of each population under each geographic treatment condition. Southern populations (SP1 and SP2) are represented in gray, central populations (UK1 and UK2) are represented in black. Statistical differences are summarized in Supporting Information Table S1

*Corallina officinalis* net production rates (Figure 3) in central populations did not change significantly in the different conditions over the course of the experiment. Contrary to central population 1, central population 2, however, showed significantly lower primary production rates under southern compared to central conditions after the experiment (*p* < 0.05, Kruskal–Wallis *H* test). Southern populations showed a similar oxygen production rate before and after the experiment under southern conditions. Central populations showed lower oxygen production rates under southern conditions but higher rates under central conditions compared to pre‐experiment measurements (Figure 3). Only southern population 2 showed a significant negative change in production rates, actually resulting in respiration rates, under central experimental conditions compared to starting conditions (*p* < 0.05, Kruskal–Wallis *H* test). When comparing populations within regions, none of the central or southern populations were significantly different within each other before the experiment. However, central populations were significantly different from southern population 1 prior to the experiment (*p* < 0.05, Kruskal–Wallis *H* test).

Respiration rates of central populations (Figure 3) did not change significantly throughout the experiment despite a positive trend under central conditions and were stable under southern conditions. Respiration rates of southern populations were stable under southern conditions, whereas they increased significantly under central compared to southern conditions (*F*_1,5_ = 25.346 and *F*_1,5_ = 21.848 for southern populations 1 and 2, respectively, *p* < 0.01, 1‐way ANOVA for both southern populations). Central populations showed a significant difference between each other after the experiment under both temperature treatments (*F*_1,5_ = 36.975 and *F*_1,5_ = 26.769 for central populations 1 and 2, respectively, *p* < 0.01, 1‐way ANOVA in both cases). Comparing populations, none of the populations within a region were significantly different from each other before the experiment. Under central as well as southern conditions, all central populations were significantly different from all southern populations (Under central conditions: *F*_1,5_ = 19.119 for UK1 vs. SP1, *F*_1,5_ = 18.837 for UK1 vs. SP2, *F*_1,5_ = 16.455 for UK2 vs. SP1, *F*_1,5_ = 16.431 for UK2 vs. SP2, *p* < 0.05, 1‐way ANOVA for all cases; under southern conditions: *F*_1,5_ = 130.367 for UK1 vs. SP1, *F*_1,5_ = 125.643 for UK1 vs. SP2, *F*_1,5_ = 61.485 for UK2 vs. SP1, *F*_1,5_ = 60.270 for UK2 vs. SP2, *p* < 0.01, 1‐way ANOVA for all cases) but not significantly different from each other.

### 3.3 Calcification

**Figure 4:**
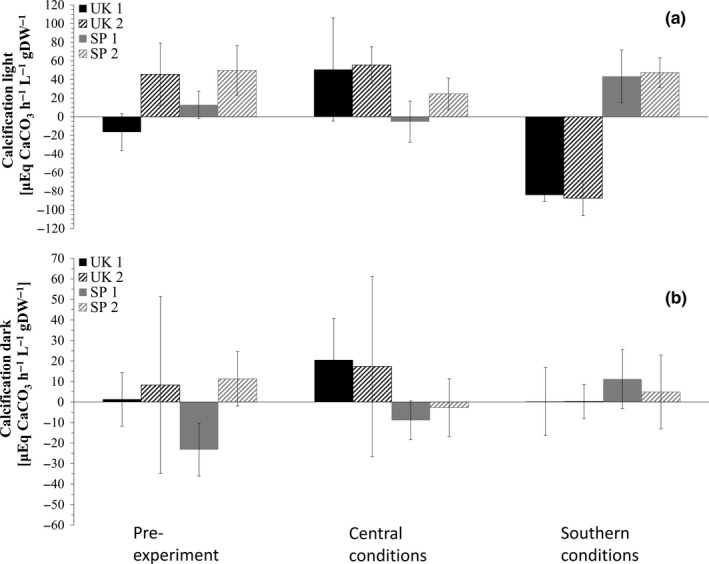
(a) Calcification rates in the light and (b) dark [μmol hr^−1^ L^−1^ gDW^−1^] ±*SD* of *Corallina officinalis* before and after the common garden experiment of each population under each geographic treatment condition. Southern populations (SP1 and 2) are represented in gray, central populations (UK1 and 2) are represented in black. Statistical differences are summarized in Supporting Information Table S1

Calcification rates in light (Figure 4) were significantly different before and after the experiment under southern conditions for southern population 1 (*p* < 0.05, Tukey HSD *p* < 0.05, 2‐way ANOVA), but not for southern population 2. Under central conditions, southern populations showed a nonsignificant decrease of calcification rates. In contrast to central population 2, central population 1 showed a significant increase in calcification rates in the light under central conditions (*p* < 0.05, Tukey HSD *p* < 0.01, 2‐way ANOVA) and both showed a highly significant lower rate under southern conditions (*p* < 0.01, Tukey HSD *p* < 0.01, 2‐way ANOVA in both cases) compared to pre‐experiment as well as the central conditions (*p* < 0.05, Tukey HSD *p* < 0.05, 2‐way ANOVA in both cases). In contrast to the second population, southern population 1 is significantly different to both central populations under central conditions (*p* < 0.05, Tukey HSD *p* < 0.05, 2‐way ANOVA in both cases). Comparing populations within a region, populations were found not to be significantly different from each other throughout the experiment.

Calcification rates in the dark (Figure 4) increased under central conditions but decreased under southern conditions for central populations. The development of dark calcification rates for southern populations showed a significant increase for population 1 under southern conditions compared to the beginning of the experiment (*F*_2,6_ = 5.013, *p* < 0.05, Tukey HSD *p* < 0.05, 2‐way ANOVA) but no significant change for southern population 2. Under central conditions, southern populations showed an increasing trend. Comparing populations within a region, only the southern populations were significantly different from each other before the experiment (*F*_1,4_ = 11.016, *p* < 0.05, 1‐way ANOVA).

## 4 DISCUSSION

Understanding the physiology of *C. officinalis* and its interactions with the environment is crucial to predict how different portions of the species distribution may be affected by current and future warming rates. We present evidence for distinct vulnerability to thermal stress of central and peripheral populations of *C. officinalis,* a key marine ecosystem engineer (Daleo, Escapa, Alberti, & Iribarne, 2006; Kelaher, Underwood, & Chapman, 2003).

This study also provides genetic evidence that *C. officinalis* is found farther south than reported by earlier studies of *C. officinalis* distribution (Williamson et al., 2015). We present Illa de Arousa (SP1) and Tragove (SP2) as the most southern, genetically confirmed populations of *C. officinalis* distribution in the Eastern North Atlantic.

Oxygen evolution curves (P‐I curves) and calcification evolution curves (C‐I curves) show clear regional responses. This suggests that data obtained in this study could be interpreted as representative data for most populations of these and surrounding regions (Egilsdottir, Olafsson, & Martin, 2015; Williamson et al., 2017), excluding those in extreme environments. However, there is added complexity when we consider the maximum level of oxygen produced (*P*_max_), indicating its dependency with the initial environment of the population. Central populations may get more stressed under higher temperature conditions, as already published for crustose coralline algae (Diaz‐Pulido, Anthony, Kline, Dove, & Hoegh‐Guldberg, 2012). *C*_max_ confirms that central conditions are more favorable than southern conditions for all four populations. In P‐I curves, southern populations show a steeper α than central populations, demonstrating a more efficient light harvesting capability. The steeply decreasing α in oxygen evolution curves of southern populations under central conditions can be explained either by increased physiological stress and a lower capacity for photosynthesis or by a geographic effect causing different responses depending on latitudinal origin of populations (Williamson et al., 2018). For the same populations and conditions however, α of the calcification evolution curves increases slightly which outline an opposite trend compared to α of oxygen evolution curves described above. This coincides with the observed, significant reduction of net production but not calcification rates for southern populations. A minimal increase of α in oxygen evolution curves of central populations under southern conditions correlates with Williamson et al. (2018) who also found that central populations do not differ significantly in winter and summer conditions, which are similar to southern conditions in this study. In warmer temperatures, the tested populations also show an increase in their saturating light irradiance (*I*_K_) for C‐I curves; however, even higher *I*_K_ values for calcification evolution have been reported in France and the UK (Egilsdottir et al., 2015; Williamson et al., 2017). This indicates that *C. officinalis* might be able to withstand future central conditions regarding *I*_K_ for its calcification, up to a certain extent. *I*_K_ values for P‐I curves are lower under southern compared to central conditions for all populations in this study. This indicates that all specimens maintained under southern conditions reach their maximum photosynthetic level at lower irradiance and could therefore experience damage of photosynthetic systems sooner compared to same populations living under central conditions. Additionally, maximum photosynthetic levels at low irradiance together with the lower light harvesting efficiency may result in exhaustion of photosynthetic systems due to very fast reactions within the metabolic pathways and the energy consumption for photon harvesting. Southern temperature conditions, therefore, present a less favorable environment for all populations.

This study highlights a significant change in primary production of southern margin and central populations when subjected to temperature conditions of the opposite distributional region. Central populations show reduced respiration rates under southern conditions. Similar behavior was found by Davison (1991) for the brown alga *Saccharina latissima*. Central populations show reduced net production under southern conditions indicating stress. However, they are still able to withstand warmer temperatures, in contrast to southern populations which may not be able to survive changing conditions over prolonged periods in their current habitat and may experience a southern range contraction (Lima, Ribeiro, Queiroz, Hawkins, & Santos, 2007). Net production rates of southern populations drop drastically under central conditions, resulting in oxygen uptake instead of production. These rates are similar to rates found under southern conditions, indicating these populations are unable or may take a longer time to adapt to central conditions. It has been shown that photosynthesis can stimulate calcification and that its increase could offset the CaCO_3_ dissolution in different calcifying algae in response to increased CO_2_ (Borowitzka, 1989; Gattuso, Allemand, & Frankignoulle, 1999; Hofmann et al. 2012; Johnson, & Carpenter, 2012; McCoy, Pfister, Olack, & Colman, 2016). Therefore, it can be expected and findings indicate that central populations downregulate calcification before photosynthesis and respiration in warmer temperature conditions.

Calcification in the light and in the dark showed drastic negative changes for central populations under southern conditions, confirming the uncoupling of photosynthesis and calcification. Central populations are shutting down light calcification metabolism resulting in dissolution. Central and southern populations have similar light calcification before and after the experiment under central control conditions demonstrating that there was no tank/experiment effect on the populations. Light calcification and dark calcification found in the literature are lower than these found in this study (Egilsdottir et al., 2013; El Haïkali, Bensoussan, Romano, & Bousquet, 2004; Williamson et al., 2017). This coincides with the elevated photosynthesis and respiration rates. Southern populations reduce light calcification only by a small amount under central conditions (McCoy et al., 2016) but show increased respiration and decreased net production, suggesting downregulation of photosynthetic activity before calcification mechanisms. This is also supported by *C*_max_ values obtained in this study which are higher under central conditions than under the control southern conditions. High variability in dark calcification was observed in all populations in all treatment, reflecting findings of McCoy et al. (2016), and indicating that the already small amount of dark calcification is easily influenced by environmental parameters such as temperature. Dark calcification rates of central populations decreased to close to zero under southern conditions, confirming downregulation of dark calcification. This, in addition to the decline in light calcification rates, results in a higher dissolution rate of central populations under southern conditions than under control conditions. A decrease of dark calcification of southern populations under central conditions indicated slow or no ability of southern populations to adapt to colder temperature regimes. At the same time, central populations show similar behavior in warmer temperatures. All of this shows that the two processes of calcification and photosynthesis are not as tightly coupled as previously assumed.

While oxygen evolution and calcification evolution data perfectly mirror the net production and respiration, calcification results show the high in situ variability between central populations which is due to environmental adaptations rather than genetic adaptations. This is supported by the very similar calcification rates in the light after the incubation of central populations in southern conditions compared to the pre‐experimental measurements. This also means that we are unable to determine the timing of when central populations kept under southern conditions began to reduce light calcification rates.

A high variability in both *α* and *I*_K_ of P‐I and C‐I curves, also found by Egilsdottir et al. (2015), in southern populations shows that adaptation ability can strongly depend on region of origin and the stress already experienced in their natural environment. Combining the knowledge gained from this study, central populations seem to be more robust, resilient, and adaptable to future climatic changes as predicted by the IPCC (2013) than southern populations. According to Williamson et al. (2017), *C. officinalis* populations in South England are adapted to variability in environmental stressors both in short (tidal) and long (seasonal) timescales. This is consistent with the findings of primary production, respiration as well as light and dark calcification, leaving the central populations better adapted to changes than southern populations in this study and others. This supports the center‐to‐margin hypothesis (Guo, Taper, Schoenberger, & Brandle, 2005) for *C. officinalis*. Additionally, this study also demonstrates that *C. officinalis* needs a minimum amount of 4 weeks, but preferably longer, to fully acclimatize to altered conditions such as changes in temperature or pCO_2_.

Even though relatively static conditions were kept in this experiment and no tidal movement was simulated due to facility restrictions, parameters were chosen to be at the predominant existing light and temperature conditions of the season in which the algae were sampled in and which they would experience when submerged during intertidal periods. This ensured the measurement of the optimum and most realistic physiological responses of *C. officinalis* to the opposite populations conditions by being in their predominant state of being submerged and not stressed by abiotic factors during low tide conditions.

The speed in which climatic changes are observed today may be too rapid for this species to be able to adapt fully to them. This may result in loss of genetic diversity in *C. officinalis,* and species ranges may shift and become confined. This was previously also found by Collevatti, Nabout, and Diniz‐Filho (2011) in a common garden experiment with the neotropical tree species *Caryocar brasiliense*, which experiences a climate change induced restriction of ideal habitat for their southern margin populations. According to the authors, this leads to a drastic decrease of genetic diversity and numbers of alleles due to the survival and reproduction success of only the most adapted genotypes under future climatic conditions. Additionally, it leads to the reduction in individual fitness and therefore population evolutionary potential. Another study performed a common garden experiment on multiple species of ants and found an elevated temperature induced decrease in survival and brood production (Pelini et al., 2012). The authors of this study indicate that the decline in these two factors will lead to a loss of genetic diversity. A potential change in genetic diversity and percentage cover due to a potential decrease in growth rates of *C. officinalis* in rocky shore intertidal ecosystems has been suggested to lead to loss of diversity of this macroalgae genus as well as associated flora and fauna. It is unclear, however, how flora and fauna would adapt to gradual changes in hosts.

To conclude, central populations may be adapting better to warmer temperature conditions in future oceans but at the same time may experience a loss in percentage cover in intertidal, rocky, coastal zones. Central populations’ calcification is negatively impacted by elevated sea surface temperatures, potentially resulting in reduced resilience. Southern populations' most negatively affected physiological process in central conditions was production rates, implying a reduced resilience in these populations as well. With sea surface temperatures warming up in northern regions of the species distribution, southern populations may be able to shift their distribution northern wards and therefore potentially disappear entirely in their original environment due to even warmer conditions in future ocean environments.

## CONFLICT OF INTEREST

The authors declare that they have no conflict of interest.

## AUTHOR CONTRIBUTIONS

RK and FR designed the research. RK performed the research. KN and GZ performed the genetic analysis. RK led the writing of the manuscript with contribution from FR, AF, KN, SM, and GZ. FR provided critical feedback and helped shape the research and analysis. All authors contributed substantially to revisions of the manuscript.

## Data Availability

Statistical data supporting the results are available under: https://doi.pangaea.de/10.1594/PANGAEA.899568.
